# A systematic review of the research on telework and organizational economic performance indicators

**DOI:** 10.3389/fpsyg.2022.1035310

**Published:** 2022-12-21

**Authors:** Jean Claude Mutiganda, Birgitta Wiitavaara, Marina Heiden, Sven Svensson, Arne Fagerström, Gunnar Bergström, Emmanuel Aboagye

**Affiliations:** ^1^Department of Business and Economic Studies, University of Gävle, Gävle, Sweden; ^2^School of Business and Economics, Discipline of Accounting and Control, Åbo Akademi University, Turku, Finland; ^3^Department of Occupational Health Sciences and Psychology, Faculty of Health and Occupational Studies, University of Gävle, Gävle, Sweden; ^4^Unit of Intervention and Implementation Research for Worker Health, Institute of Environmental Medicine, Karolinska Institutet, Stockholm, Sweden

**Keywords:** telework, employee turnover, systematic review, organizational economic performance, employee perceived performance

## Abstract

**Introduction:**

A systematic review is conducted in the study to investigate the relationship between telework and organizational economic performance indicators such as self-reported employee performance, organizational performance, actual employee turnover rates, or intentions.

**Methods:**

The databases Scopus, Business Source Premier, and Web of Science were used to conduct a literature search. Original articles published from 2000 and up to May 2021 were selected. Studies were screened for inclusion independently by review pairs and data were extracted. The Mixed Methods Appraisal Tool (MMAT) was used to evaluate the quality of the studies included.

**Results:**

Forty-three studies were included with some addressing multiple outcomes. Self-reported performance was higher for teleworking employees compared to those working in the ordinary workplace. The extent of the change in performance was dependent on individual characteristics and the extent of the teleworking practice in the organization. Telework was also associated with increased organizational performance, particularly in homogenous samples with unique work tasks. When telework is voluntary, it appears that both actual employee turnover rates and intentions to leave the organization are lower.

**Discussion:**

Further research with high-quality prospective designs is necessary to properly understand the contribution of telework to organizational economic performance indicators.

## Introduction

Teleworking refers to working in a place outside the ordinary workplace where time flexibility or not can occur (Allen et al., 2015). Teleworking is a component of remote work practices, providing employees with greater freedom to alternate between the ordinary workplace and outside locations, mostly *via* the use of information and communications technology (ICT). While not a new phenomenon, teleworking increased significantly during the COVID-19 pandemic when organizations implemented intensive home-based teleworking in response to the global COVID-19 lockdowns and other pandemic related restrictions (EU-OSHA, 2021). Significant human resource management difficulties, including, but not limited to, where people should perform their tasks in small and large organizations occurred during the pandemic. Home-based teleworking was highly recommended for employees who could work remotely from home under the COVID-19 pandemic.

Before the pandemic struck, most organizations and employees were largely unprepared for shifts toward teleworking. Over half of EU workers lacked any prior teleworking experience (EU-OSHA, 2021). According to the Eurofund (2020), telework is most common in the Scandinavian countries, accounting for 38 and 33% of the workforce in Denmark and Sweden, respectively. Other EU countries with a high proportion of teleworkers include the Netherlands (31%), Luxembourg (29%), the United Kingdom (27%), France (26%), and Estonia (25%). This shows that telework agreements are more popular in the north and west of Europe, however there are notable outliers, such as Germany, with 13% below the EU average, and Estonia, with 24% above it. The data also showed variation in teleworking rates by occupation and socio-occupational category since some occupations are not suited to telework, for instance those in construction, hospitality, and personal services. When the COVID-19 pandemic spread widely rapidly, organizations whose work could be done from the outside of the regular workplace implemented broad use of telework to keep their business operations running while avoiding the virus's spread at work (Eurofund, 2020).

Prior to the pandemic, many employees had formal or informal agreements with their employers to work from home or another location. During the pandemic, much changed, resulting in a shift from direct presence or face-to-face supervision of work to full-time telework forms in which most work functions were conducted *via* technology or platform-based ICT in many businesses (Eurofund, 2021). Whether these changes would have occurred “organically” if COVID-19 had not broken out, and whether these changes will remain post COVID-19, especially now that the restrictions have been removed remains an open question in tele-workable sectors and occupations. An increasing number of organizations are debating whether to continue with teleworking, such as home-based telework or other hybrid teleworking forms i.e., part-time in the office, part-time at home or some other location (Neeley, 2021). However, there is limited empirical research on the question of what teleworking means for organizational economic performance indicators i.e., outcomes that are measured and managed by organizations because they are important to their success. Previous studies provide ambiguous insight into organizational economic performance indicators for employees and organizations and therefore does not help management to understand whether telework makes economic sense and how it can be embedded in appropriate human resources management practices. An understanding of what telework implies for management is critical to ensuring that any future, more permanent modifications to teleworking regulations benefit both employees and the organization.

Teleworking is generally linked to several metrics of importance to the organization's bottom line namely, employee performance and productivity, absenteeism, turnover, commitment, and overall organizational performance (Bailey and Kurland, 2002; Tietze et al., 2009; de Menezes and Kelliher, 2011; Allen et al., 2015; Kazekami, 2020). From previous research, the relationship may be positive yet inconclusive on employees' perceptions and other performance reports (Samek Lodovici, 2021). In previous reviews, there was little unambiguous proof that telework increased organizational financial outcomes, yet teleworking is generally considered to promote productivity, decrease turnover, and improve organizational performance (Bailey and Kurland, 2002; Gajendran and Harrison, 2007; Harker Martin and MacDonnell, 2012). As in previous reviews, the evidence from de Menezes and Kelliher (2011) did not demonstrate a business case for the use of flexible work arrangements (FWAs). According to de Menezes and Kelliher (2011), employees in FWAs may have access to a variety of flexible or non-standard work arrangements, such as choice over when work is completed, work away from the ordinary workplace, working full-time hours in fewer days, or reduced work hours. Some studies argue that a more inclusive approach to employee and organizational outcomes, as well as comparison groups, gender issues, different appreciation of workspace and time, and high-quality methodological designs, are necessary to make sense of the contradictory evidence of organizational economic performance outcomes attributable to telework alone (Tietze et al., 2009; De Ruiter and Peters, 2022). This suggests that knowledge of FWAs i.e., work away from the ordinary workplace and whom it works for, and in what circumstances the practice works including different categories of occupations and individual workers characteristics, is needed. This review presents up-to-date knowledge based on high-quality studies about how telework is associated with organizational economic performance outcomes.

The purpose of this study is to compile and synthesize the findings of previous studies on the relationship between telework and organizational financial outcomes in terms of self-reported employee performance, organizational performance, actual employee turnover rates or intentions. The review seeks to answer two main research questions: (1) How is telework related to employees' self-reported measures such as work or job performance, productivity, work content execution, effectiveness, turnover intentions, etc.? and (2) How is telework related to objective organizational performance indicators including sales, added value, actual turnover, productivity, etc.?

The two primary contributions of this study are as follows: (1) Using data from relatively high-quality research, this review study assesses the evidence of an association between telework and productivity based on employees' self-reported performance or organizational records as well as actual turnover or intentions considering variations between businesses. The review study provides a comprehensive review that focuses on varied teleworking arrangements and the consequences on different organizational financial outcomes. (2) Because of the thorough information provided in some of the original research, the review study identifies some of the probable factors that are associated with organizational financial losses due to telework by occupation, albeit some of these factors may be shared by all occupations.

## Materials and methods

### Study design

A systematic review was conducted, which is a step-by-step approach to synthesizing the findings of multiple primary research studies (Fink, 2019). This systematic review study adheres to the preferred reporting guideline for systematic review and meta-analysis (PRISMA) guidelines (Page et al., 2021).

### The PEO framework

The PEO (i.e., population, exposure, and outcome) framework was used for the present search. The PEO as a framework can be especially useful when investigating the prospects of developing a certain outcome because of an exposure, as well as assist in focusing the review process and identifying searchable parts of a research question (Schardt et al., 2007).

#### Population

The population consisted of individuals working in organizations whose working arrangements for employees included flexible work locations. As a result, studies investigated included employees working in organizations who are allowed to work in a place outside the ordinary workplace (such as home-based telework or virtual or distant or remote work, where time flexibility or not can occur). Studies which had investigated organizational-level outcomes in relation to flexible work location practices were also included in this review.

#### Exposure

This definition of telework arrangement is used—a work practice that involves members of an organization substituting a portion of their typical work hours (ranging from a few hours per week to nearly full-time) to work away from the ordinary workplace—principally from home—using technology to interact with others as needed to conduct work tasks (Shockley and Allen, 2007). Central to the definition is that work can be performed outside of the traditional temporal and/or spatial boundaries of the ordinary workplace (including full-time work from home but not necessarily limited to home-based work) and includes work from home-based businesses. Because of the nature of the exposure under consideration, this review covers research with a variety of designs, including intervention studies.

#### Outcome

Organizational economic performance indicators investigated in this study include financial performance (when referring to return on investments or profitability, cost-saving practices) and performance indicators (when referring to self-reported performance, productivity, and turnover). The term performance is of high economic interest to organizations and can be measured in terms of perceived actual or potential increase or decrease in work output i.e., employees' perception of their own performance, or in relation to their colleagues' or the employer's assessment. For some organizations, the actual or the potential performance on a specific task at the individual level are aggregated at the team and/ or organizational level to represent productivity or the value created from the resources available (Tangen, 2005). Employee turnover which refers to employees leaving the organization must be lowered to keep acceptable performance levels. Performance and employee turnover can be major weapons for organizations to achieve cost and quality advantages over their competitors (Tangen, 2005).

### Literature search

**Figure 1:**
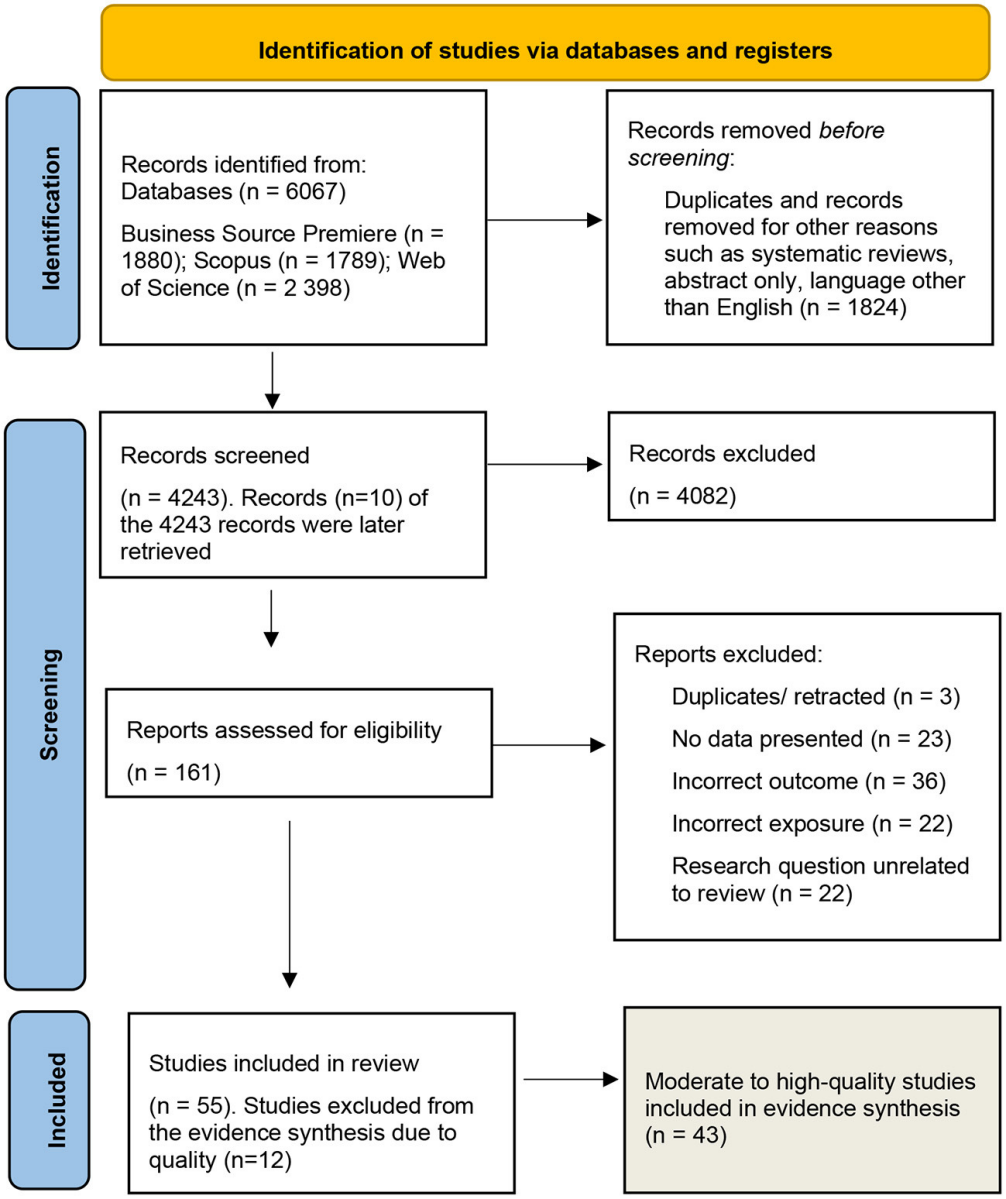
PRISMA flowchart of study selection process.

Together with an information specialist, we formulated a systematic, documented literature search strategy to identify relevant literature based on the PEO framework. The search was conducted in two waves in collaboration with an information specialist. The first was a test search, which was performed in November 2020, aiming to identify, refine, and focus the search terms. The test search was performed in six databases: Scopus, PubMed, Emerald, Business Source Premier, Academic Search Elite, and Web of Science. The second search in May 2021 was a final search conducted across three databases: Scopus, Business Source Premier, and Web of Science. These three databases were preferred because they are multidisciplinary and cover a wide range of research fields, they allow for free-text searches, and they provide access to some of the databases used in the first search. The literature search covered studies published from 2000 through and until May 2021. The search string is available in the Supplementary material 1. The search resulted in a total of *n* = 6,067 articles. After excluding duplicates, a total of 4,239 articles were left to be examined (Figure 1).

### Study records

All relevant studies were compiled in Endnote or Mendeley reference managers. The records were saved in PDF format for full text reading and subsequent quality assessment, as well as to permit independent screening and cataloging of discrepancies amongst reviewers.

### Inclusion and exclusion

The main criteria for inclusion and exclusion of literature which were defined in advance were as follows:

1. The population of the study should be clearly described and relevant, i.e., the research should concern organizations whose working arrangements allow work from a different location than the employer's workplace through ICT and the employees working in such organizations. Self-employed workers or business owners were not included.
2. The exposure investigated should be clearly described, measured and relevant, i.e., the working conditions in which it is allowed for a degree of flexibility and interaction between workers doing their work tasks that can be performed outside of the ordinary workplace context, including but not limited to home-based work or remote work.
3. Studies that examined non-specific collective concepts such as “flexible work arrangements” or unspecified workplace were not considered relevant as it is difficult to assess what the actual work location is in such cases.
4. Studies that focus only on the traditional temporal flexibility such as flextime and organizational practice of functional flexibility that requires employees to work from the central office were excluded.
5. The investigated outcome should be clearly described, measured and relevant, i.e., including but not limited to financial performance (such as return on investments or profitability, cost-saving practices e.g., rent cost reductions, sales, etc.) and non-financial performance indicators (such as self-reported performance, employer quality assessment productivity, organizational-level performance, and turnover).
6. The study should examine the link between telework practice and organizational economic outcomes at the employee or organizational level.
7. The study should be an original study, published in English, peer-reviewed, containing quantitative or qualitative data and published between 2000 and May 2021 in a scientific journal.
8. Knowledge summaries and systematic reviews, as well as theoretical articles that did not analyze their own data, opinions, study protocols, articles that only contained abstracts, student dissertations, and other gray literature, were all excluded.

### Study selection

The assessment of relevance of the articles obtained from the systematic literature search was carried out in two selection rounds, based on the predefined inclusion and exclusion criteria. The first selection round was based on the article's title and abstract. The full text of articles that were considered relevant were read through in the next selection round to determine whether they were relevant to include in the subsequent quality assessment. In total of 4,243 articles were evaluated using the title and abstract. There was *n* = 10 of the 4,243 articles that could not be retrieved at first, so these were sought, and all were eventually found for inclusion in the screening process. Following the screening, *n* = 4,082 records were eliminated, leaving *n* = 161 for further consideration. Of the *n* = 161 reports evaluated for eligibility, *n* = 106 were excluded due to the following reasons: duplicates (*n* = 3), no data presented (*n* = 23), incorrect outcome described (*n* = 36), incorrect exposure described (*n* = 22), and research question posed was unrelated to review (*n* = 22). The *n* = 55 articles left after exclusion were split in three portions, where two researchers in each group read the articles separately, and thereafter discussed their evaluation to reach agreement on inclusion (see Figure 1). In the results section, only moderate to high-quality studies were included in evidence synthesis (*n* = 43).

Each article was evaluated by two researchers independently. The individual researcher's assessment was hidden from each study until two researchers had evaluated it, so that the researchers were not initially affected by each other's assessments. This ensures independent assessments of each article. After full text evaluation, *n* = 55 articles were considered to meet the inclusion criteria. These were then quality assessed.

### Data extraction and quality assessment

Ideally, data extraction should be completed in duplicate by two independent reviewers. In this review study, however, it was not practical. Thus, one reviewer extracted data, and another independently verified the results for accuracy and completeness. Based on the review objectives and research question, the data extraction and synthesis were carried out using rigorous processes that facilitate transparency of reporting on the characteristics of the included studies.

Two researchers independently assessed the quality of each article. The Hong et al. (2018) version of the Mixed Methods Appraisal Tool (MMAT) was used to assess the methodological quality of the included articles. The MMAT is a methodological quality appraisal tool that is designed for the quality assessment stage of systematic mixed studies reviews, i.e., reviews that include qualitative, quantitative, and mixed methods studies. It allows for the evaluation of the methodological quality in five categories: qualitative research, randomized controlled trials, non-randomized studies, quantitative descriptive studies, and mixed methods studies.

The MMAT contains two screening questions for all sorts of research designs to identify whether a study is empirical and hence the MMAT may be used. Based on study design type for each included study, the appropriate category of studies to appraise is chosen and a rating of criteria “Yes,” “No,” or “Can't tell.” The “Can't tell” response category means that the paper does not report adequate information to answer “Yes” or “No.” In this review, the reviewers agreed to convert “Can't tell” response category to “No,” since no information or inadequate information were provided in the study.

The MMAT discourages reviewers from calculating an overall score from the ratings of each criterion since an overall score may not always be informative. Instead, it suggests providing a more detailed presentation of the ratings of each criterion to better inform the quality of the included studies. For instance, the quality of the study can be described in stars (^*^) or percentages (%). For example, if a study receives five stars on each criterion, it could be interpreted as 100%, four stars equal 80%, three stars equal 60%, two stars equal 40%, and one star equals 20% quality criteria met. For this review study, the results of the appraisal were interpreted using arbitrary categories to help description of study quality. This study used three categories (i.e., low, medium, and high) to clearly describe included studies. Studies with five stars (one star for each criterion) were assessed to be of high quality, while those with three to four stars were of moderate quality and those with two stars or less were of low quality.

## Results

This review includes a total of 55 articles, with several studies containing more than one outcome. Four of the 55 studies were performed across countries, with the rest coming from different countries, with U.S. (20 studies), Australia (five studies), the U.K. (five studies), Japan (three studies), and China (two studies). Canada, Germany, Finland, Ireland, Portugal, Spain, Italy, Belgium, South Africa, and Iran each had one study. The vast majority (67%, *n* = 37) were published between 2015 and 2021. The research covered private companies and public organizations in the banking and manufacturing sectors, information technology, healthcare and life insurance, government agencies, travel agencies, and other knowledge-intensive occupations. More than half (60%, *n* = 33) investigated perceived employee performance, 29% (*n* = 16) investigated objective organizational performance indicators, and 18% (*n* = 10) investigated actual employee turnover and turnover intentions. In the Supplementary material 2: Characteristics of the included studies on telework and their comparator are provided. For details on the number of studies divided into study population, key outcome measure, findings, and quality assessment for the different study designs, see Tables 1–**3**. In the Supplementary material 3: The quality assessment ratings (i.e., the ratings of each criterion of MMAT) for the final 55 studies are provided. The next sections present the findings from studies of moderate to high quality (*n* = 43).

**Table 1:** Findings from studies on telework and self-reported performance or productivity.

**References**	**Population/organization type**	**Key outcome measure**	**Findings**	**Rating**
**Quantitative randomized controlled trials**				
Sherman (2020)	Abcam PLC life sciences company, England. *n* = 187 employees.	Employees' job performance	Telework improve job performance especially for mothers	*****
**Quantitative non-randomized studies**				
Bao et al. (2022)	Large IT firm, China. Four thousand records of *n* = 107 developers.	Productivity e.g., the number of builds/commits/code reviews	Developers Working from home have similar productivity to those working onsite.	***
Delanoeije and Verbruggen (2020)	Construction and property development firm, Belgium. *n* = 78 (39 each in intervention and control group)	Person-and day-level job performance	No differences in person-level performance, but day-level performance was higher for telework users.	*****
De Menezes and Kelliher (2016)	Four organizations in the professional sector, e.g., pharmaceutical, banking, etc., UK. *n* = 2,617 employees	Individual performance	Remote working has positive indirect effects on performance.	***
Feng and Savani (2020)	US resident fulltime employees, USA. *n* = 286 fulltime employees	Perceived work productivity	Women's perceived work productivity dropped when working from home during the COVID-19 pandemic	****
Gajendran et al. (2015)	Employers and employees, a wide assortment of organizations, USA. *n* = 323 employees and *n* = 143 matched supervisors	Task performance	Telecommuting positively associated with task performance	*****
Golden et al. (2008)	Large high-tech company, USA. A matched sample of *n* = 261 professional-level teleworkers and their managers	Job performance	Extensive teleworking in isolation negatively impacts performance	****
Golden et al. (2008)	Large high-tech company, USA. *n* = 375 professional-level virtual employees	Job performance	Extensive virtual mode workers have higher job performance	****
Golden and Gajendran (2018)	Supervisors and employees, a single organization, USA. *n* = 273 telecommuters and their supervisors	Job performance	Telecommuting had a positive association with job performance	****
Hill et al. (2003)	IBM, USA. Traditional office, *n* = 4,316, virtual office, *n* = 767, and home office, *n* = 441	Job performance, productivity, workload success	Virtual/home office appear to positively impact performance	***
Hyland et al. (2005)	Eight private and public organizations, Ireland. *n* = 172 employees from different organizations	Employee performance	Telework had no connection with performance	***
Kitagawa et al. (2021)	Four chemical and automobile manufacturing companies, Japan. *n* = 22,815 employees	Perceived productivity	Home-based work leads to a productivity decline	*****
Medina-Garrido et al. (2017)	Employees of banking sector, Spain. *n* = 1,511 employees	Job performance	Flexi-place indirectly related to performance through wellbeing	***
Morikawa (2020)	RIETI Survey of Corporate Management and Economic Policy, Japan. *n* = 3,324 sample was mainly used	Perceived productivity	Home-based work productivity was lower during the COVID-19	****
Narayanamurthy and Tortorella (2021)	Multiple organization sectors, UK. *n* = 106 employees	Employees' performance (i.e., output quality and delivery)	Home-based office enhances output quality and delivery	***
Ralph et al. (2020)	Survey of software developers, multi-country study. *n* = 2,225 usable responses from 53 countries.	Perceived productivity	Lower perceived productivity from home-based work	****
Tsukamoto (2021)	Survey of workers in the general population, Japan. *n* = 908 respondents	Productivity	Telecommuting leads to higher productivity	***
van der Lippe and Lippenyi (2020)	Survey of nine EU countries, EU. *n* = 869 teams and 11,011 employees from 259 establishments	Task performance, individual and team	Home-based work negatively impacts coworker performance	****
Vega et al. (2014)	U.S. government organization, USA. *n* = 180 employees	Job performance	Teleworkers report higher levels of job performance	***
**Quantitative descriptive studies**				
Aguilera et al. (2016)	SMEs, France. *n* = 940 responses from representative sample of residents of the Brittany	Perceived productivity	No association between home-based work and perceived productivity	***

### Telework and perceived productivity/job performance

A total of 20 studies examined the relationship between teleworking and employees' or managers perceived productivity and/or job performance (Table 1). Except for one randomized study (Sherman, 2020), almost all the studies described in this section were quantitative descriptive or non-randomized i.e., mostly descriptive, or analytical cross-sectional studies.

Generally, supervisors and employees who could voluntarily work from home rated their perceived performance higher than those who worked from the employer's premises. Studies conducted on home-based office during the COVID-19 pandemic show perceived work productivity drop during the COVID-19 pandemic (Feng and Savani, 2020; Morikawa, 2020; Kitagawa et al., 2021). Sherman (2020) randomized study, conducting analysis for different subgroups, shows that teleworking enhanced job performance considerably for most subgroups, with female employees (mothers) benefiting the most. In their quasi-experimental study, Delanoeije and Verbruggen (2020), the users of telework reported slightly higher day-level performance on teleworking days but there were no significant differences in person-level performance between the users and non-users of telework.

Nine studies with cross-sectional designs (Hill et al., 2003; Golden and Veiga, 2008; Vega et al., 2014; Gajendran et al., 2015; De Menezes and Kelliher, 2016; Medina-Garrido et al., 2017; Golden and Gajendran, 2018; Narayanamurthy and Tortorella, 2021; Tsukamoto, 2021) found that telework was positively associated with high productivity or better job performance. However, telework was shown not to be associated with any substantial improvement in productivity or job performance in three studies (Hyland et al., 2005; Aguilera et al., 2016; Bao et al., 2022). In van der Lippe and Lippenyi (2020) study, the findings show that when more coworkers work from home, employee and team performance can be negatively impacted, but team performance tends to deteriorate the most. This implies that when coworkers do not work from home, team performance appears to improve, pointing to the interconnections of group and individual tasks. Informally negotiated remote working practice or access to flexi-place had positive indirect effects on employee performance through commitment, and job satisfaction (De Menezes and Kelliher, 2016; Medina-Garrido et al., 2017).

In unpacking the role of voluntary teleworkers' job characteristics, studies investigating social job characteristics such as job interdependence, social support, and superior-subordinate relationships in an extensive telework mode found high levels of job performance in low levels of interdependence, low levels of social support, and high quality superior-subordinate relationships than employees who worked a limited amount in telework mode (Golden and Veiga, 2008; Golden et al., 2008; Golden and Gajendran, 2018). Knowledge job characteristics such as job complexity and problem solving show a positive relationship between telework and job performance, but most importantly, the extent of telework explained job performance, which ranged from benign to positive (Golden and Veiga, 2008; Golden et al., 2008; Golden and Gajendran, 2018).

### Telework and objective organizational performance indicators

Seven out of 15 studies (Kitou and Horvath, 2007; Patti, 2014; Bloom et al., 2015; Ruostela et al., 2017; Choudhury et al., 2020; Zhang et al., 2021), showed positive benefits of telework on objective organizational performance (Table 2). According to Bloom et al. (2015) findings, worker productivity rose in the telework group compared to the control group without affecting the level of quality of work. Choudhury et al. (2020) study exploiting a natural experiment found that working form anywhere as opposed to home resulted in an increase in employee output, with no increase in rework. However, according to their model, all telework programs, whether from home or anywhere, increase productivity incrementally when compared to working in the office. In Zhang et al. (2021) study, small businesses performed better overall in states with higher work-from-home rates when industry-specific variations were considered, along with local economic, demographic, and policy factors. In Giovanis (2018) study, using an instrumental variable approach in a prospective design, responses from the management or their representatives indicated a significant positive relationship between telework and financial and labor performance.

**Table 2:** Findings from studies on telework and objective organizational performance/ productivity.

**References**	**Population/organization type**	**Key outcome measure**	**Findings**	**Rating**
**Quantitative randomized controlled trials**				
Bloom et al. (2015)	Travel agency, China. *n* = 249 randomized call center employees	No. of phone calls	Home working led to performance increases	*****
**Quantitative non-randomized studies**				
Choudhury et al. (2020)	Patent and Trademark Office, USA. *n* = 831 patent examiners	Total actions, rework	Work from anywhere resulted in increase in the total number of actions	****
Giovanis (2018)	Management, random workplaces, G. Britain Panel data set for workplaces, with ~11,500–16,000 observations	Workplace performance; two alternative measures financial performance and labor productivity.	Positive relationship between telework and performance	*****
Kotey and Sharma (2019)	Public, private, and non-profit organizations, Australia. *n* = 4,204 employees	Return on labor	Work from home reduced return on labor	*****
Lee and Hong (2011)	Federal agencies, USA. *n* = 105 employees	Proportion of met or exceeded annual performance indicators	Telework has a negative association with performance	****
Monteiro et al. (2021)	Large Portuguese firms (>250 employees), Portugal. 4,726 firm-year observations	Sales per employee	Working remotely is more likely to be harmful for productivity	****
Neirotti et al. (2012)	Different Italian firms from industry groups, Italy. *n* = 1,134 companies included.	Value added per employee	Home-based telework do not exhibit higher labor productivity than “mobile work”	****
Patti (2014)	Health and Life Insurance Company, USA *n* = 342 Insurance processors and examiners and *n* = 45 managers	No. of claims processed and examined	Teleworking increased productivity and lowered office expenses	****
Rocha et al. (2021)	Firms in Cyprus, Georgia, Greece, Italy, Moldova, and Russian Federation. *n* = 3,864 firms included	Sales growth	No overall statistically significant effect of telework, more positive effect on firms with greater growth	***
Ruostela et al. (2017)	Managers, salespeople and consultants in a production company, Finland *n* = 52 employees	Space usage, occupancy costs, environmental impact	New ways of working are cost saving and improves environmental performance	****
St George et al. (2009)	Department of Human Services, Australia. *n* = 13 telenursing call operators	Quality of advice, risk incidents, no. of phone calls	Working from home is positive for no. phone calls and had no statistically significant effect on quality and risk incidents	*****
Viete and Erdsiek (2020)	German service firms, Germany *n* = 1,045 observations	Sales	Work from home did not statistically significant affect sales.	***
Zhang et al. (2021)	Survey of small businesses, USA. *n* = 8,399 observations	Operating revenue, disruption of supply chain, business closures, cash flows	Higher home-based work rates positively influence operating revenue, disruption of supply chain and cash flow, no effect on business closures	*****
**Quantitative descriptive studies**				
Kitou and Horvath (2007)	Simulated scenarios based on national data, USA. Simulated data from the *n* = 81 literature and surveys	Energy and fuel costs, external costs related to air emissions	Telework programs reduce energy and fuel costs in the office space	****
KlindŽić and Marić (2019)	Large-sized organizations, Croatia. *n* = 171 organizations, HR managers surveyed	Return on assets, return of equity, revenue per employee	No statistically significant effect of telework or home-based work	*****

A few of the studies that show positive results on organizational indicators also investigated outcomes like space usage, occupancy costs, fuel and energy costs, and environmental costs. The studies found that as telework programs and frequency increased, environmental performance might improve, which would benefit businesses by lowering workplace costs (Kitou and Horvath, 2007; Ruostela et al., 2017).

Four studies found negative impacts of telework on organizational performance (Lee and Hong, 2011; Kotey and Sharma, 2019; Neirotti et al., 2019; Monteiro et al., 2021). In the Lee and Hong (2011) study, telework programs performed significantly worse than other family-friendly initiatives like childcare subsidies, paid leave for caregiving, and flexible work schedules. Kotey and Sharma (2019) study found that working from home has a direct negative association with return on labor. According to the Neirotti et al. (2012) study, organizations that use telecommuting practices and operate in more dynamic business environments while also adopting a higher rate of information systems observe productivity gains compared with labor productivity when teleworking from home. This suggests that home-based telework is less productive than the type of teleworking that involve telecommuting strategies. The Monteiro et al. (2021) study found that, except for R&D organizations, where working remotely benefits the organization in terms of performance indicators, there is a significantly negative association between remote access and productivity. Four studies found that telework was not related with any significant gain in organizational performance (St George et al., 2009; KlindŽić and Marić, 2019; Viete and Erdsiek, 2020; Rocha et al., 2021).

### Telework and intentions to leave/stay or actual turnover rates

Eight studies examined the association between different aspects of telework and intentions to leave or actual turnover rates (Table 3). Two of the studies had longitudinal designs (Caillier, 2016; Choi, 2020), one quasi-experiment design (Lee and Kim, 2017) and the remainder had a descriptive or analytical cross-sectional designs (Hyland et al., 2005; Golden, 2006; Caillier, 2011; Masuda et al., 2012; Dilmaghani, 2021).

**Table 3:** Findings from studies on telework and intentions to leave or actual turnover rates.

**References**	**Population/organization type**	**Key outcome measure**	**Findings**	**Rating**
**Quantitative non-randomized studies**				
Caillier (2011)	Federal Government employees, USA. *n* = 263,475 federal government employees	Dichotomous (considering leaving organization within the next year, yes/no)	Teleworkers and non-teleworkers reported similar intentions to quit	****
Caillier (2016)	Federal Government employees, USA. *n* = 144 observations from 36 agencies	Actual turnover rates	Telework had no impact actual turnover	***
Choi (2020)	Federal Government employees, USA. *n* = 428 observations from 143 sub-agencies of federal government	Voluntary turnover (register data)	Higher proportions of teleworkers reduced the rates of voluntary turnover.	*****
Dilmaghani (2021)	Canadian General Social Survey, Canada. *n* = 7,446 observations from nationally representative data	Dichotomous (considering leaving organization within the next year, yes/no)	Female teleworkers had lower turnover intentions	***
Hyland et al. (2005)	Eight public and private organizations, Ireland *n* = 172 employees from different organizations	Turnover intentions	A weak, non-significant, positive association between telework and turnover intentions	***
Lee and Kim (2017)	Federal Government employees, USA. *n* = 194,739 federal employees	Intention to stay	Telework eligibility has positive association with intention to stay	*****
Masuda et al. (2012)	Managers of organizations, 15 countries (Asian, American, and Latin American country clusters) *n* = 3,918 managers from 15 countries	Turnover intentions	No association between telework and turnover intentions.	***
Golden (2006)	Large internet solution corporation, USA *n* = 393 employees	Turnover intentions	More teleworking weakened turnover intentions.	***

In Lee and Kim (2017), telework eligibility had a positive association with intention to stay. Dilmaghani (2021) study found no differences in the intentions to leave between male teleworkers and non-teleworkers, but female workers who teleworked in addition to having access to flexible working hours were less likely to consider changing jobs the following year compared to those who only teleworked. Golden (2006) found a weak, yet significant, negative association between the proportion of telework time per week and turnover intentions fully mediated by exhaustion. This study suggests that telework might reduce work exhaustion, which in turn reduce intentions to leave. According to Hyland et al. (2005) and Masuda et al. (2012), employees who frequently use telework and have a strong preference for segmented work and home roles showed a weak positive correlation with turnover intentions. Two studies using data from different years within the same organizational context, i.e., the US federal government, found that telework, or satisfaction with the potential to telework either had no impact on actual turnover intentions or reduced it (Caillier, 2016; Choi, 2020). Two of the studies found that teleworkers and non-teleworkers reported similar intentions to quit or no association between telework availability/eligibility and turnover intentions (Caillier, 2011).

## Discussion

This review searched and analyzed the body of existing research to clarify the relationship between telework and critical self-reported and objective economic performance indicators at the individual and organizational level.

In general, employees and managers who could choose to telework rated their perceived performance higher than those who were required to work on the employer's premises, but to a differing extent. Employees working from home appear generally to have higher levels of self-reported job performance and productivity (Tsukamoto, 2021), as well as perform better on an objective creative assignment (Vega et al., 2014), than those working in an office. However, different types of work-family policies, such as flexible work location (flexi-place), may be indirectly related to employee performance mediated by employee wellbeing (Medina-Garrido et al., 2017), family-work conflict (Sherman, 2020), social interactions with managers and family members (Neufeld and Fang, 2005), employee preference for work segmentation (Hyland et al., 2005), and virtual connection technologies (Narayanamurthy and Tortorella, 2021).

Similarly, depending on the prevailing work-related circumstances and characteristics of the employees, type and size of the task, telework could be perceived differently as either having positive or negative associations with performance (Bao et al., 2022). This suggests that the performance metric used by studies varied considerably, which results in diverse findings among the studies included. Further, research on telework during the COVID-19 pandemic found a perceived decline in work productivity. Employees perceived that they were less productive during the COVID-19 pandemic, which could be expected considering the lack of childcare, inadequate technology, and other amenities (Ralph et al., 2020).

In this review, studies indicated beneficial impacts of telework on organizational performance typically among homogenous samples (e.g., call center operators) with unique work tasks (St George et al., 2009; Patti, 2014; Bloom et al., 2015; Choudhury et al., 2020). Studies that showed negative or no impact of telework, on the other hand, were more likely to cover different types of organizations and rely on more general organizational economic performance measures. In the study by Monteiro et al. (2021), which found both negative (small firms) and positive (R&D firms) association between remote access (as a proxy for telework) and sales, it was suggested that the association depended on the type of activities performed by the organizations. For instance, small businesses did not engage in exporting and hired workers with lower levels of skill. Similarly, Zhang et al. (2021) study reported a substantial variation in the effect of home-based work across industry sectors. Hence, there is not uniformity in the literature with respect to factors associated with productivity in home-based or teleworking organizations (OECD, 2020). The different conclusions arrived at by the studies might not be caused by the type of activity only. There could be reasons such as nature of work (Boell et al., 2016), technology availability (OECD, 2020), industry type (Monteiro et al., 2021), tasks (Bao et al., 2022), sufficient communication with colleagues and managerial support (Coenen and Kok, 2014), and other social-health-psychological factors such as commuting time and interruptions (Kazekami, 2020), social and professional isolation (Felstead and Henseke, 2017), affecting employees in different ways, which can negatively impact employee and organizational productivity.

According to the studies reviewed, using telework or being eligible to telework could determine whether employees stayed with the company or left it (Caillier, 2016; Choi, 2020). Although the conclusions were fairly consistent, most of the findings showed weak and non-significant associations from studies with methodological issues, such as evaluating data without taking into account people who are nested in multiple countries and/or organizations (Masuda et al., 2012); using non-random sampling or cross-sectional designs in which exposure and result were gathered simultaneously. Further, although some studies clearly state that turnover rate is defined as the number of employees who left the company during the year divided by the average number of employees over that time multiplied by 100, it is unclear whether it was voluntary leave, involuntary leave, temporary hires, or temporary leaves that were used in the estimate. Some work and employee characteristics that influence intentions to quit or stay include employees who were denied the opportunity to telework, i.e., no eligibility to telework, despite their personal preferences for segmented work and telework (Hyland et al., 2005), the amount of telework time per week (Golden, 2006), and home roles due to gender (Hyland et al., 2005; Dilmaghani, 2021). This suggests that it is the possibility to choose the optimal mix of telework and office hours based on one's preferences, rather than teleworking *per se*, which motivates employee's intentions to stay or leave the organization. More studies in different work contexts are required to confirm these associations.

Home-based or hybrid telework might have had implications for organizational productivity during the pandemic. According to Batut and Tabet (2020), during the pandemic, home-based or hybrid teleworking was heavily reliant on high-quality supervision and managerial support (e.g., by providing ICT infrastructure or training, ergonomics), which were critical for positive teleworking experiences and productivity. The findings of this review suggest that individual and organizational outcomes in telework were not only associated with the supportive management style (Choi, 2020), and type of job/ industry (Zhang et al., 2021; Bao et al., 2022), but also the work set-up and experience of employees during the pandemic (Morikawa, 2020; Rocha et al., 2021; Tsukamoto, 2021). As earlier pointed out, some studies conducted during the pandemic indicated negative association of telework with individual and organizational outcomes, such as, self-rated performance (Feng and Savani, 2020; Mirela, 2020; Morikawa, 2020; Kitagawa et al., 2021), organizational performance (Ralph et al., 2020; Monteiro et al., 2021), and employee turnover (Dilmaghani, 2021). Other studies also conducted during the pandemic found no significant change in individual and organizational outcomes due to telework (Chapman and Thamrin, 2020; Dixit et al., 2020; Moretti et al., 2020; Viete and Erdsiek, 2020). More studies on telework supervision and management, type of job/industry, telework intensity before and after the pandemic might better contribute to the understanding of difference in organizational economic performance indicators.

### Telework influences on organizational policies and practices

Telework has become a solution for people at different stages in their lives, when they may be studying, bringing up a family, or growing older, or it can simply match their individual preferences by letting them decide when and where to work. Employees seem to be willing to choose this form of work since it improves their working and social lives by easing work constraints and yielding gains in autonomy over their own affairs. However, there are pros and cons, in particular the cost-benefit trade-off for organizations and employees practicing telework (Golden, 2001).

Likely, widespread teleworking in the long-term has implications for self-reported performance, productivity, and intention to stay or leave the organization. Working outside of the ordinary workplace may be challenging for both employee and organization, especially in the aftermath of the COVID-19 pandemic. Many high-profile businesses want to accept this flexible work future to attract employees, and many employees are striving to spend as little time in the workplace as possible—and others are planning to leave employers who are averse to working from anywhere, at least for now. To maximize the gains inherent in the use of more widespread telework from the perspective of the employer and employee, organizations could promote investments in its physical apparatus (i.e., workspace, ICT, and home office ergonomics) and enhance the relation between managers and employees who choose this work form. Uninterrupted ICTs are critical in allowing employees who prefer to telework from home or anyplace to engage in work activities (Eurofund, 2020). In the post-COVID-19 era, targeted public policies related to productivity gains from teleworking can be beneficial to both private and public organizations (OECD, 2020). Public policies and co-operation among social partners (i.e., employers, employees, and other stakeholders) are crucial to ensure that new, efficient, and welfare-improving working methods emerging after the pandemic can be developed and maintained as conventional forms of telework practices.

A comparison of how different work venues (e.g., traditional office, virtual office, and home office) influence aspects of work and organizational outcomes were considered in the reviewed studies. The review findings suggest that there is a potential for continual teleworking in terms of self-reported performance and organizational economic performance indicators, which could be obtained from the best combinations of different flexible working arrangements. For instance, Yamashita et al. (2022) observed impaired work functioning among employees who preferred and teleworked four or more days a week compared with those who almost never teleworked. Although this review did not investigate closely the topic of frequency or intensity and preference for telework, it would be interesting to study whether frequency/intensity of telework in relation to preference for it has any significance for organizational performance. Organization (i.e., either private or public) may need to evaluate their employees' needs to be flexible and accommodating, especially if they wish to recruit and retain a diverse workforce by finding the sweet spot of flexible working arrangements combinations.

### Strength and limitations of the review

This review has several advantages. The systematic review process allows for a qualitative description of included studies to uncover gaps and provides a basis for clear findings through a thorough search of existing published literature on the topic. This review is based on the findings of studies moderate to high quality studies. Low-quality studies were excluded from the evidence synthesis. This notwithstanding would not affect the conclusion drawn for employee turnover and self-reported performance outcomes. There were no low-quality studies on the objective outcome of organizational performance. In comparison to previous reviews, this article addresses a broader range of employee and organizational outcomes, gender issues, and different perceptions of traditional temporal and/or spatial work practices, allowing for a more nuanced assessment of the relationship between telework and organizational economic performance outcomes. However, some limitations of the study should be mentioned. Most of the included studies were cross-sectional non-experimental study designs, precluding inferences of causality. Thus, the studies do not provide information on whether telework is the cause of performance/productivity changes or decision to stay on the job or leave. To our knowledge, only a few studies have adopted a true experimental methodology in a field setting and have found positive effects of telework on turnover intentions and work performance (Bloom et al., 2015; Sherman, 2020). Many studies also lack generalizability. It may be difficult, for example, to generalize findings from a study of younger employees to older employees, or to generalize findings from certain organizations, because the organization type determines how performance can be measured and the tasks performed in the different organizations differ. This is especially important in job performance research since various work performance levels fluctuate with industry type. The studies' methodological limitations, as well as substantial heterogeneity in organizations and work tasks, definitions of telework as well as comparison work forms, the different ways organizational outcomes were measured, complicate a general, overall conclusion. This implies that the quality of evidence on the relationships between organizational economic outcomes and telework should be interpreted reasonably.

After selecting all relevant studies, the critical and constructive analysis of the quality of the studies were performed using the MMAT. The MMAT is a critical appraisal tool that was developed for use in systematic mixed studies reviews (i.e., reviews combining qualitative, quantitative and/or mixed methods studies). The MMAT has been criticized for not being thorough enough for evaluating mixed methods studies (O'Cathain, 2010), however other reviews of critical appraisal tools found the opposite (Crowe and Sheppard, 2011). In the present study, we excluded 12 studies with low quality (i.e., two stars or less). Although the cut-off points for low, moderate, and high quality were arbitrary, they were valuable for qualitative assessment, and we have described in detail how the appraisal results were interpreted and applied in the review.

## Conclusion

Several studies found that telework is associated with increased perceived job performance and organizational performance particularly in homogenous samples with unique work tasks. When telework is voluntary, it appears that both actual employee turnover rates and intentions to leave the organization are lower. Further research on the implementation and evaluation of effective work forms including but not limited to home-based telework and hybrid telework is needed to understand their contribution to self-rated performance and organizational economic performance indicators. High-quality prospective studies are clearly needed in the future. This effort will contribute to the knowledge on how to organize and implement such working arrangements in a way that is beneficial and sustainable for employees, organizations, and society.

## Data availability statement

The original contributions presented in the study are included in the article/Supplementary material, further inquiries can be directed to the corresponding author.

## Author contributions

EA contributed to formal analysis, visualization, writing—original draft preparation, supervision, and project administration. All the authors agree to be accountable for the content of the work, contributed to data curation, screening, and selection of articles for this review, and contributed to conceptualization, methodology, investigation, resources, and writing—review and editing. All authors contributed to the article and approved the submitted version.
