# Supplementary material for: A systematic review of the research on telework and organizational economic performance indicators
Source: Front Psychol. 2022 Dec 21;13:1035310. doi: 10.3389/fpsyg.2022.1035310 (PMC9812566; doi:10.3389/fpsyg.2022.1035310)
Supplement: Supplementary file 1 [file Data_Sheet_1.PDF]

Supplementary Table SA1: Search string on organizational economic performance indicators and telework

Scopus			
No	Description		Items found (approx.)
1	Flexible work	TITLE-ABS-KEY(flextime OR flexitime OR flexplace OR "flexible work*" OR "flexible work* arrangements" OR "flexible work* hours" OR "work* flexibility" OR "staggered hours of labor" OR "variable work* hours" OR "flexible hours" OR "flexible schedul*" OR "flexible work*" OR telecommut* OR "home offices" OR "home work*" OR "work* from home" OR "work* at home" OR "home-based offices" OR "home-based work*" OR "homebased work*" OR telework* OR "remote work*" OR "distance work*" OR "virtual work*" OR "new ways of working" OR "virtual offices" OR "virtual work teams" OR "workplace flexibilit*" OR "work-place flexibilit*" OR "work place flexibilit*" OR "distributed work arrangements" OR "distributed work*" OR "alternat* work schedules" OR "variable work* hours" OR "alternat* work* arrangements" OR "remote employment" OR "virtual work* teams" OR "home labor" OR "Modern work*" OR homework* OR "home work*" OR "new ways of working")	36 874
2	Indicators	TITLE-ABS-KEY ("optimization" OR "cost efficiency" OR "value based management" OR "value-based management" OR "economic value of man" OR "human capital" OR "human assets" OR "human resources" OR "social responsibility of business" OR "social responsibility of industry" OR "generally accepted accounting principles" OR "financial accounting standards" OR "accounting" OR "accountancy" OR "generally accepted auditing standards" OR "economic value added" OR productivity OR "output controls" OR "management outcomes"	3 037 262

		OR profit* OR (indicators W/0 (business OR financial OR economic OR leading OR "key success" OR performance OR "key performance")) OR (Value W/0 (creation OR capture OR "capture of" OR added OR "based management" OR firm OR enterprise OR organi?ational OR company OR corporate)) OR (accounting W/0 (financial OR commercial OR socio-economic OR socioeconomic OR standards OR "human resource" OR social OR firm OR enterprise OR organi?ational OR corporate)) OR (audit* W/0 (policies OR standards OR principles)) OR (efficiency W/0 (managerial OR company OR corporate OR industrial OR firm OR organi?ational OR firm OR enterprise)) OR (organi?ational OR corporate OR firm OR enterprise OR company W/0 (behavior OR ambidexterity OR goals OR performance OR objectives OR effectiveness OR outcomes)) OR (corporate OR organi?ational OR firm OR enterprise OR company W/0 ("social accounting" OR "social responsibility reporting" OR citizenship OR responsibility OR responsibility OR "social accountability" OR "social responsibility" OR sustainability OR goals OR effectiveness OR accountability)) OR (performance W/0 (business OR company OR corporate OR metrics OR financial OR firm OR enterprise OR "product development")))	
3		1 AND 2	3 813
4		3 AND Filters activated: Publicationtype: artiklar Language: english, svenska, danska, norska, finska, undefined Time: 2000-<br><br>Reviewer = 127	1 789

**Web of Science**			
No	Description		Items found (approx.)

1	Flexible work	TS=(flextime OR flexitime OR flexplace OR "flexible work*" OR "flexible work* arrangements" OR "flexible work* hours" OR "work* flexibility" OR "staggered hours of labor" OR "variable work* hours" OR "flexible hours" OR "flexible schedul*" OR "flexible work*" OR telecommut* OR "home offices" OR "home work*" OR "work* from home" OR "work* at home" OR "home-based offices" OR "home-based work*" OR "homebased work*" OR telework* OR "remote work*" OR "distance work*" OR "virtual work*" OR "new ways of working" OR "virtual offices" OR "virtual work teams" OR "workplace flexibilit*" OR "work-place flexibilit*" OR "work place flexibilit*" OR "distributed work arrangements" OR "distributed work*" OR "alternat* work schedules" OR "variable work* hours" OR "alternat* work* arrangements" OR "remote employment" OR "virtual work* teams" OR "home labor" OR "Modern work*" OR homework* OR "home work*" OR "new ways of working")	22 003
2	Indicators	TS=("optimization" OR "cost efficiency" OR "value based management" OR "value-based management" OR "economic value of man" OR "human capital" OR "human assets" OR "human resources" OR "social responsibility of business" OR "social responsibility of industry" OR "generally accepted accounting principles" OR "financial accounting standards" OR "accounting" OR "accountancy" OR "generally accepted auditing standards" OR "economic value added" OR productivity OR "output controls" OR "management outcomes" OR profit* OR (indicators NEAR0 (business OR financial OR economic OR leading OR "key success" OR performance OR "key performance")) OR (Value NEAR0 (creation OR capture OR "capture of" OR added OR "based management" OR firm OR enterprise OR organi?ational OR company OR corporate)) OR (accounting NEAR0 (financial OR commercial OR socio-economic OR socioeconomic OR standards OR "human resource" OR social OR firm OR enterprise	2 374 545

		OR organi?ational OR corporate)) OR (audit* NEAR0 (policies OR standards OR principles)) OR (efficiency NEAR0 (managerial OR company OR corporate OR industrial OR firm OR organi?ational OR firm OR enterprise)) OR (organi?ational OR corporate OR firm OR enterprise OR company NEAR0 (behavior OR ambidexterity OR goals OR performance OR objectives OR effectiveness OR outcomes)) OR (corporate OR organi?ational OR firm OR enterprise OR company NEAR0 ("social accounting" OR "social responsibility reporting" OR citizenship OR responsibility OR responsibility OR "social accountability" OR "social responsibility" OR sustainability OR goals OR effectiveness OR accountability)) OR (performance NEAR0 (business OR company OR corporate OR metrics OR financial OR firm OR enterprise OR "product development")))	
3		1 AND 2	3 585
4		3 AND Filters activated: Publicationtype: artiklar Language: english, svenska, danska, norska, finska, unspecified Time: 2000-<br><br>Reviewer = 91	2 398

**EBSCO – Business source premier**			
No	Description		Items found (approx.)
1	Flexible work	TI(flextime OR flexitime OR flexplace OR "flexible work*" OR "flexible work* arrangements" OR "flexible work* hours" OR "work* flexibility" OR "staggered hours of labor" OR "variable work* hours" OR "flexible hours" OR "flexible schedul*" OR "flexible work*" OR telecommut* OR "home offices" OR "home work*" OR "work* from home" OR "work* at home" OR "home-based offices" OR "home-based work*" OR "homebased work*" OR telework* OR	29 567

		"remote work*" OR "distance work*" OR "virtual work*" OR "new ways of working" OR "virtual offices" OR "virtual work teams" OR "workplace flexibilit*" OR "work-place flexibilit*" OR "work place flexibilit*" OR "distributed work arrangements" OR "distributed work*" OR "alternat* work schedules" OR "variable work* hours" OR "alternat* work* arrangements" OR "remote employment" OR "virtual work* teams" OR "home labor" OR "Modern work*" OR homework* OR "home work*" OR "new ways of working")<br><br>OR<br><br>AB(flextime OR flexitime OR flexplace OR "flexible work*" OR "flexible work* arrangements" OR "flexible work* hours" OR "work* flexibility" OR "staggered hours of labor" OR "variable work* hours" OR "flexible hours" OR "flexible schedul*" OR "flexible work*" OR telecommut* OR "home offices" OR "home work*" OR "work* from home" OR "work* at home" OR "home-based offices" OR "home-based work*" OR "homebased work*" OR telework* OR "remote work*" OR "distance work*" OR "virtual work*" OR "new ways of working" OR "virtual offices" OR "virtual work teams" OR "workplace flexibilit*" OR "work-place flexibilit*" OR "work place flexibilit*" OR "distributed work arrangements" OR "distributed work*" OR "alternat* work schedules" OR "variable work* hours" OR "alternat* work* arrangements" OR "remote employment" OR "virtual work* teams" OR "home labor" OR "Modern work*" OR homework* OR "home work*" OR "new ways of working")<br><br>OR<br><br>DE(flextime OR "flexible work arrangements" OR telecommunting OR "virtual offices" OR "home offices" OR "virtual work teams" OR "home labor")	

2	Indicators	TI("optimization" OR "cost efficiency" OR "value based management" OR "value-based management" OR "economic value of man" OR "human capital" OR "human assets" OR "human resources" OR "social responsibility of business" OR "social responsibility of industry" OR "generally accepted accounting principles" OR "financial accounting standards" OR "accounting" OR "accountancy" OR "generally accepted auditing standards" OR "economic value added" OR productivity OR "output controls" OR "management outcomes" OR profit* OR (indicators N0 (business OR financial OR economic OR leading OR "key success" OR performance OR "key performance")) OR (Value N0 (creation OR capture OR "capture of" OR added OR "based management" OR firm OR enterprise OR organi?ational OR company OR corporate)) OR (accounting N0 (financial OR commercial OR socio-economic OR socioeconomic OR standards OR "human resource" OR social OR firm OR enterprise OR organi?ational OR corporate)) OR (audit* N0 (policies OR standards OR principles)) OR (efficiency N0 (managerial OR company OR corporate OR industrial OR firm OR organi?ational OR firm OR enterprise)) OR (organi?ational OR corporate OR firm OR enterprise OR company N0 (behavior OR ambidexterity OR goals OR performance OR objectives OR effectiveness OR outcomes)) OR (corporate OR organi?ational OR firm OR enterprise OR company N0 ("social accounting" OR "social responsibility reporting" OR citizenship OR responsibility OR responsibility OR "social accountability" OR "social responsibility" OR sustainability OR goals OR effectiveness OR accountability)) OR (performance N0 (business OR company OR corporate OR metrics OR financial OR firm OR enterprise OR "product development")))<br><br>OR	2 869 272

		AB("optimization" OR "cost efficiency" OR "value based management" OR "value-based management" OR "economic value of man" OR "human capital" OR "human assets" OR "human resources" OR "social responsibility of business" OR "social responsibility of industry" OR "generally accepted accounting principles" OR "financial accounting standards" OR "accounting" OR "accountancy" OR "generally accepted auditing standards" OR "economic value added" OR productivity OR "output controls" OR "management outcomes" OR profit* OR (indicators N0 (business OR financial OR economic OR leading OR "key success" OR performance OR "key performance")) OR (Value N0 (creation OR capture OR "capture of" OR added OR "based management" OR firm OR enterprise OR organi?ational OR company OR corporate)) OR (accounting N0 (financial OR commercial OR socio-economic OR socioeconomic OR standards OR "human resource" OR social OR firm OR enterprise OR organi?ational OR corporate)) OR (audit* N0 (policies OR standards OR principles)) OR (efficiency N0 (managerial OR company OR corporate OR industrial OR firm OR organi?ational OR firm OR enterprise)) OR (organi?ational OR corporate OR firm OR enterprise OR company N0 (behavior OR ambidexterity OR goals OR performance OR objectives OR effectiveness OR outcomes)) OR (corporate OR organi?ational OR firm OR enterprise OR company N0 ("social accounting" OR "social responsibility reporting" OR citizenship OR responsibility OR responsibility OR "social accountability" OR "social responsibility" OR sustainability OR goals OR effectiveness OR accountability)) OR (performance N0 (business OR company OR corporate OR metrics OR financial OR firm OR enterprise OR "product development")))<br><br>OR<br><br>DE("corporate sustainability" OR "economic indicators" OR "value-based management"	

		OR "value creation" OR "value capture" OR "value added (Marketing)" OR "economic value added (Corporations)" OR "accounting" OR "accounting standards" OR "auditing standards" OR "auditing policies" OR "human capital" OR "human resource accounting" OR "social accounting" OR "social responsibility of business" OR "benefit corporations (Business structure)" OR "organizational performance" OR "key performance indicators (Management)" OR "organizational effectiveness" OR "organizational goals" OR "industrial efficiency" OR "organizational ambidexterity" OR "organizational behavior" OR "NEW product development" OR "OPERATIONS management" OR "RESOURCE management" OR "PERSONNEL management" OR "INDUSTRIAL productivity" OR "PRODUCTIVITY accounting" OR "INDUSTRIAL management" OR "INDUSTRIAL efficiency" OR "PRODUCTIVITY accounting" OR "PRODUCTION (Economic theory)" OR "PRODUCTIVITY measurement"))	
3		1 AND 2	8 251
4		TI("Systematic Review*" OR "Systematic literature eview*" OR "systematic literature analysis" OR "systematically review" OR "systematic synthesis" OR "meta synthesis" OR "metasynthesis" OR "qualitative synthesis" OR "meta ethnography" OR "metaethnography" OR "meta study" OR "metastudy" OR "meta summary" OR "metasummary" OR "thematic synthesis" OR "critical interpretive synthesis" OR "grounded theory synthesis" OR "meta interpretation" OR "cross case analysis" OR "scoping review*" OR "realist review*" OR "evidence map*" OR "mapping review*" OR "systematic mapping" OR "rapid review*" OR "literature review*")<br><br>OR<br><br>AB("Systematic Review*" OR "Systematic literature eview*" OR "systematic literature	26 773

		analysis" OR "systematically review" OR "systematic synthesis" OR "meta synthesis" OR "metasynthesis" OR "qualitative synthesis" OR "meta ethnography" OR "metaethnography" OR "meta study" OR "metastudy" OR "meta summary" OR "metasummary" OR "thematic synthesis" OR "critical interpretive synthesis" OR "grounded theory synthesis" OR "meta interpretation" OR "cross case analysis" OR "scoping review*" OR "realist review*" OR "evidence map*" OR "mapping review*" OR "systematic mapping" OR "rapid review*" OR "literature review*")<br><br>OR<br><br>DE("Literature Reviews" OR "meta analysis" OR "state of the art reviews")	
5		3 NOT 4	8 189
6		3 AND Filters activated: Publicationtype: Academic journals, Scholarly (peer reviewed) journals; Language: english, svenska, danska, norska Time: 2000-	1 880

frontiers
