# Supplementary material for: A systematic review of the research on telework and organizational economic performance indicators
Source: Front Psychol. 2022 Dec 21;13:1035310. doi: 10.3389/fpsyg.2022.1035310 (PMC9812566; doi:10.3389/fpsyg.2022.1035310)
Supplement: Supplementary file 2 [file Data_Sheet_2.PDF]

Supplementary Material

Supplementary Table SA2: Characteristics of the included studies on telework and their comparator

**Author**	**Intervention/ Exposure**	**Comparator**
Hill et al., 2003	Virtual office, home office	Traditional office
Hyland et al., 2005	Telework	Flextime, compressed workweeks
Golden, 2006	Teleworking	
Golden et al., 2008	Virtual mode	
Golden et al., 2008	Teleworking	
Kitou et al., 2008	Telework	Nontelework scenarios
St George et al., 2009	Working from home	Working in the centre
Lee et al., 2011	Telework	Childcare subsidies, paid leave for family care, alternative work schedules
Masuda et al., 2012	Telecommuting	Part-time, Flex time, Compressed work week
Neirotti et al., 2012	Home-based telework	mobile forms of telework, nonadopters
Caillier, 2011	Telework	Non-teleworkers
Patti, 2014	Work from home	
Vega et al., 2015	Telework	Non-teleworkers

Bloom et al., 2015	Work from Home	Traditional office
Gajendran et al., 2015	Telecommuting	
Ruostela et al., 2015	New ways of working (Mobile, Flex, Fixed)	Old office
Caillier, 2016	Telework	Alternative work schedules, health and wellness programs, employee assistance programs, childcare, elder care
Aguilera et al., 2016	Home-based/satelite office telework	Traditional office
Delanoeije et al., 2020	Users of telework	Non-users of telework
De Menezes et al., 2017	Formal and informal remote working arrangement	No flexibility or flexibility in working hours
Medina-Garrido et al., 2017	Flexi-place	Flexi-time, long leaves
Giovanis, 2018	Home-based working, teleworking	flexible timing and compressed hours
Lee et al., 2018	Telework	Non-teleworkers
Kotey et al., 2019	Work from Home	Flex Start, Job Share, Part-time, Time in lieu of overtime (TOIL), Flexible Leave, Banking hours
Klindžić et al., 2019	Employee-driven practice (telework, home-based work)	Employer driven practice (shift, annual hours, casual, etc.)
Golden et al., 2019	Telecommuting	
Choudhury et al., 2020	Work from-anywhere (WFA), Working from home	Working in the office

Sherman, 2020	Remote working	Working in the office
Choi, 2020	Teleworking	
Ralph et al., 2020	Work from home	
van der Lippe et al., 2020	Work from home	
Morikawa, 2020	Work from home	Usual workplace
Viete et al., 2020	Work from home	Trust-based worktime, working time accounts
Feng et al., 2020	Work from home	
Monteiro et al., 2021	Remote electronic access	
Tsukamoto, Y. 2021	Experienced teleworkers	Inexperienced teleworkers, Work at office
Zhang et al., 2021	Work from Home	
Bao, 2021	Work from home	Working on site
Kitagawa et al., 2021	Work from home	Working on site
Dilmaghani, 2021	Flexplace	Flextime, both flextime and flexplace
Narayanamurthy et al., 2021	Remote working	
Rocha et al., 2021	Remote working	
