# Supplementary material for: A systematic review of the research on telework and organizational economic performance indicators
Source: Front Psychol. 2022 Dec 21;13:1035310. doi: 10.3389/fpsyg.2022.1035310 (PMC9812566; doi:10.3389/fpsyg.2022.1035310)
Supplement: Supplementary file 3 [file Data_Sheet_3.PDF]

Supplementary Material

Supplementary Tables SA3: The quality assessment ratings using the MMAT (n=55 studies)

Studies on telework and self-reported performance or productivity

		**Criteria from the Mixed Methods Appraisal Tool**																										
**Studies**	**Study design**	1.1	1.2	1.3	1.4	1.5	2.1	2.2	2.3	2.4	2.5	3.1	3.2	3.3	3.4	3.5	4.1	4.2	4.3	4.4	4.5	5.1	5.2	5.3	5.4	5.5	Quality	% criteria met
Bosua et al., 2013	Qualitative research	No	No	No	No	No																						
Chapman et al., 2020	Quantitative non-randomized studies											No	No	No	No	Yes											*	20%
De Menezes et al., 2017	Quantitative non-randomized studies											No	Yes	No	Yes	Yes											***	60%
Giovanis, 2018	Quantitative non-randomized studies											Yes	Yes	Yes	Yes	Yes											*****	100%
Illegems et al., 2004	Quantitative non-randomized studies											No	No	No	No	Yes											*	20%
Martínez Sánchez et al., 2008	Quantitative non-randomized studies											No	Yes	No	No	Yes											**	40%
Sherman, 2020	Quantitative randomized controlled trials						Yes	Yes	Yes	Yes	Yes																*****	100%
Vega et al., 2015	Quantitative non-randomized studies											Yes	No	Yes	No	Yes											***	60%
Collins, 2005	Quantitative non-randomized studies											No	No	No	No	Yes											*	20%
Hill et al., 2003	Quantitative non-randomized studies											Yes	No	No	Yes	Yes											***	60%
Aboalmaali et al., 2015	Quantitative descriptive studies																Yes	No	No	No	No						*	20%
Aguilera et al., 2016	Quantitative descriptive studies																Yes	Yes	No	Yes	No						***	60%
Gajendran et al., 2015	Quantitative non-randomized studies											Yes	Yes	Yes	Yes	Yes											*****	100%
Golden et al., 2008	Quantitative non-randomized studies											No	Yes	Yes	Yes	Yes											****	80%
Golden et al., 2008	Quantitative non-randomized studies											No	Yes	Yes	Yes	Yes											****	80%
Ralph et al., 2020	Quantitative non-randomized studies											No	Yes	Yes	Yes	Yes											****	80%
Tsukamoto, 2021	Quantitative non-randomized studies											No	Yes	Yes	No	Yes											***	60%
van der Lippe et al., 2020	Quantitative non-randomized studies											No	Yes	Yes	Yes	Yes											****	80%
Bao, 2021	Quantitative non-randomized studies											No	Yes	Yes	No	Yes											***	60%
Hopkins et al., 2019	Quantitative descriptive studies																No	No	No	No	yes						*	20%
Mirela, 2020	Quantitative descriptive studies																No	No	No	No	No							
Dixit et al., 2020	Quantitative descriptive studies																No	No	No	No	No							
Golden et al., 2019	Quantitative non-randomized studies											Yes	Yes	No	Yes	Yes											****	80%
Feng et al., 2020	Quantitative non-randomized studies											Yes	Yes	No	Yes	Yes											****	80%
Torten et al., 2016	Quantitative non-randomized studies											No	No	No	No	Yes											*	20%
Moretti et al., 2020	Quantitative non-randomized studies											No	No	No	No	Yes											*	20%
Conradie et al., 2019	Quantitative non-randomized studies											No	Yes	No	No	Yes											**	40%
Medina-Garrido et al., 2017	Quantitative non-randomized studies											No	Yes	No	Yes	Yes											***	60%
Narayanamurthy et al., 2021	Quantitative non-randomized studies											No	Yes	No	Yes	Yes											***	60%
Delanoeije et al., 2020	Quantitative non-randomized studies											Yes	Yes	Yes	Yes	Yes											*****	100%
Hyland et al., 2005	Quantitative non-randomized studies											No	Yes	No	Yes	Yes											***	60%
Morikawa, 2020	Quantitative non-randomized studies											No	Yes	Yes	Yes	Yes											***	80%
Kitagawa et al., 2021	Quantitative non-randomized studies											Yes	Yes	Yes	Yes	Yes											*****	100%

Studies on telework and objective organizational performance/ productivity.

		Criteria from the Mixed Methods Appraisal Tool																										
**Studies**	**Study design**	1.1	1.2	1.3	1.4	1.5	2.1	2.2	2.3	2.4	2.5	3.1	3.2	3.3	3.4	3.5	4.1	4.2	4.3	4.4	4.5	5.1	5.2	5.3	5.4	5.5	Quality	% criteria met
Choudhury et al., 2020	Quantitative non-randomized studies											No	Yes	Yes	Yes	Yes											****	80%
Kotey et al., 2019	Quantitative non-randomized studies											Yes	Yes	Yes	Yes	Yes											*****	100%
Monteiro et al., 2021	Quantitative non-randomized studies											Yes	Yes	Yes	Yes	No											****	80%
Bloom et al., 2015	Quantitative randomized controlled trials						Yes	Yes	Yes	Yes	Yes																*****	100%
Klindžić et al., 2019	Quantitative descriptive studies																Yes	Yes	Yes	Yes	Yes						*****	100%
Patti, 2014	Quantitative non-randomized studies											Yes	Yes	Yes	No	Yes											****	80%
Ruostela et al., 2015	Quantitative non-randomized studies											Yes	Yes	Yes	No	Yes											*****	80%
St George et al., 2009	Quantitative non-randomized studies											Yes	Yes	Yes	Yes	Yes											*****	100%
Zhang et al, 2021	Quantitative non-randomized studies											Yes	Yes	Yes	Yes	Yes											*****	100%
Kitou et al., 2008	Quantitative descriptive studies																Yes	Yes	Yes	No	Yes						****	80%
Lee et al., 2011	Quantitative non-randomized studies											No	Yes	Yes	Yes	Yes											****	80%
Viete et al., 2020	Quantitative non-randomized studies											No	Yes	No	Yes	Yes											***	60%
Neirotti et al., (2012	Quantitative non-randomized studies											Yes	Yes	No	Yes	Yes											****	80%
Rocha et al., 2021	Quantitative non-randomized studies											No	Yes	No	Yes	Yes											***	60%

Studies on telework and intentions to leave or actual turnover rates.

		Criteria from the Mixed Methods Appraisal Tool																										
**Studies**	**Study design**	1.1	1.2	1.3	1.4	1.5	2.1	2.2	2.3	2.4	2.5	3.1	3.2	3.3	3.4	3.5	4.1	4.2	4.3	4.4	4.5	5.1	5.2	5.3	5.4	5.5	Quality	% criteria met
Caillier, 2016	Quantitative non-randomized studies											No	Yes	No	Yes	Yes											***	60%
Martínez Sánchez et al., 2008	Quantitative non-randomized studies											No	Yes	No	No	Yes											**	40%
Choi, 2020	Quantitative non-randomized studies											Yes	Yes	Yes	Yes	Yes											*****	100%
Golden, 2006	Quantitative non-randomized studies											No	Yes	No	Yes	Yes											***	60%
Masuda et al., 2012	Quantitative non-randomized studies											No	Yes	No	Yes	Yes											***	60%
Caillier, 2011	Quantitative non-randomized studies											Yes	Yes	No	Yes	Yes											****	80%
Lee et al., 2018	Quantitative non-randomized studies											Yes	Yes	Yes	Yes	Yes											*****	100%
Hyland et al., 2005	Quantitative non-randomized studies											No	Yes	No	Yes	Yes											***	60%
Illegems et al., 2001	Quantitative non-randomized studies											No	No	No	No	Yes											*	20%
Dilmaghani, 2021	Quantitative non-randomized studies											Yes	No	No	yes	Yes											***	60%
